# Alteration of pituitary and hypothalamic membrane fluidity as a non-specific mode-of-action for reproductive effects with octamethylcyclotetrasiloxane

**DOI:** 10.17179/excli2025-8545

**Published:** 2025-10-21

**Authors:** Robert G. Meeks, James M. McKim, Jeffrey Pregenzer, Jeremy A. Durham, Debra A. McNett

**Affiliations:** 1 Shin-Etsu Silicones of America, Akron, OH, 44305, USA; 2 Lifenet Health, Life Sciences, Kalamazoo, MI, 49008, USA; 3 Dow Chemical Company, Midland, MI, 48674, USA

**Keywords:** octamethylcyclotetrasiloxane, membrane fluidity, non-specific mode-of-action, reproductive toxicity, LH surge

## Abstract

Octamethylcyclotetrasiloxane (D4) is a highly volatile cyclic siloxane used to produce silicone polymers. D4 has been shown to attenuate the LH surge in rats, resulting in reduced litter sizes. However, it has been hypothesized that these biological effects observed only at high dose levels of D4 may be because of changes in membrane microviscosity (fluidity) leading to a non-specific mode of action. Here, we set out to determine if D4 increases membrane microviscosity and link this to membrane domain function alterations. The studies reported here support the hypothesis that D4 affects ovulation via a concentration-dependent, physical-chemical mode of action that is not specific for any particular component of the neuro-endocrine system and is, therefore, not endocrine disruption but a non-specific effect. Furthermore, D4 also increases the membrane fluidity of the hypothalamic cell membrane in vitro. It is expected that a similar response would occur in vivo. This alteration in membrane fluidity decreases the release of GnRH and kisspeptin. GnRH and kisspeptin are necessary for the pre-ovulatory LH surge from the pituitary. In the absence of a GnRH and kisspeptin release, there is no signal to the pituitary for the driver of the LH surge. D4 can change membrane fluidity in vitro and likely in vivo and associated behaviors of membrane proteins/lipoproteins of various kinds via non-specific mechanisms.

## Introduction

**Figure 1:**
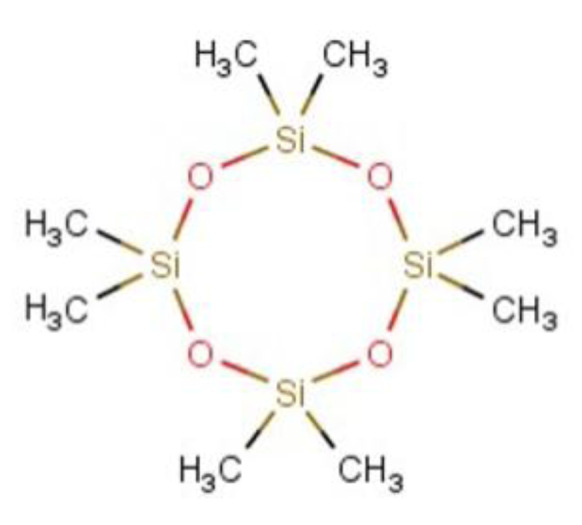
Structure of Octamethylcyclotetrasiloxane (D4)

Octamethylcyclotetrasiloxane (D4) is a cyclic siloxane of four siloxane units (Figure 1(Fig. 1)). It is a volatile, colorless, and odorless liquid of low molecular weight and water solubility. D4 is used to synthesize siloxane polymers. Residual D4 may also be present in small amounts in personal and household care silicone polymer products and may be intentionally used in industrial solvents or cleaning products. The use in manufacturing and its potential presence in various applications give rise to the potential for human exposure. Although dermal exposure is the most likely exposure route for consumers, testing by inhalation exposure (the second most prevalent route for consumers and the first exposure route for workers) is warranted because of the technical challenges associated with the dermal route of exposure due to spreading and evaporative characteristics of D4 as well as the similarity in kinetic behavior between these two exposure routes (Sarangapani et al., 2003[24]).

Effects of inhalation exposure to D4 in rat reproductive toxicity studies included a reduction in the number of corpora lutea, a decrease in litter size resulting from a reduction in the number of implantation sites in the F_0 _and F_1_ generations, prolonged estrus cycles, and decreased mating and fertility indices in the F_1_ generation (Siddiqui et al., 2007[27]). The effects were attributed to exposure in females but not males (Meeks et al., 2007[18]). Meeks et al. (2007[18]) also demonstrated that the short period around ovulation was the critical exposure period for this reproductive effect. Follow-up studies suggest inhibiting the pre-ovulatory surge of luteinizing hormone (LH) is a predominant factor (Quinn et al., 2007[22]). 

Plant et al. (2012[21]) have proposed that D4's attenuation of the LH surge in female rats is irrelevant to humans. A dopamine-like related mode of action (MoA) was considered for reproductive outcomes following inhalation exposure to D4 (Dekant et al., 2017[11]). After a weight-of-evidence (WoE) review of the results from a series of *in vivo* and in* vitro* studies to evaluate the ability of D4 to stimulate/block prolactin release *in vivo* and from specific cells *in vitro* and an evaluation of D4's affinity for dopamine receptors, the authors concluded that it is unlikely for D4 to interact directly with dopamine receptors (Dekant et al., 2017[11]). Still, they suggested that if dopamine effects were observed, post-receptor events are more likely to be the mechanism by which this occurs. These post-receptor events are often related to non-specific membrane fluidity changes.

It is possible that there is no specific molecular initiating event and that reproductive outcomes are secondary to a non-specific effect or toxicity following inhalation exposure to D4 at high concentrations. However, both could lead to non-specific impacts on gonadotropin-releasing hormone (GnRH) neuron activity and modulation of the mid-cycle GnRH surge required for the rat's mid-cycle LH release and ovulation.

It has been suggested that alterations in membrane fluidity can affect the activity of many types of signaling pathways, including those involved in LH secretion. For example, changes in fluidity may affect the diffusion of ligands and receptors involved in regulating LH secretion, potentially attenuating the LH surge. Andersen (2022[2]) concluded that the MoA for the reproductive toxicity of D4 in female rats with the strongest support is a combination of non-specific membrane fluidity changes and enhanced Gamma-Butyric Acid type a (GABAa) related disruption of GnRH neurons either in the hypothalamus or in afferent pathways affecting hypothalamic GnRH neurons. These changes would modulate the mid-cycle GnRH surge required for the rat's mid-cycle LH release and ovulation. Several authors have shown that alterations in membrane fluidity can impact cellular functions, leading to non-specific effects or indirectly to toxicity (Meeks et al., 1981[19]; Jetten et al., 1981[15], 1982[14]; Crews et al., 1983[10]). 

This paper summarizes experiments conducted to test the hypothesis proposed by Andersen (2022[2]) that at high concentrations, D4 affects reproduction by altering the physical-chemical properties of membranes. That alteration of membrane fluidity leads to GABA_a_-related disruption of GnRH neurons, either in the hypothalamus or in afferent pathways that affect hypothalamic GnRH neurons that modulate the mid-cycle GnRH surge required for mid-cycle LH release and ovulation in the rat. We performed experiments to evaluate the effects of D4 on membrane fluidity in several cell lines, including rat primary pituitary and hypothalamic cell lines. 

## Materials and Methods

### Controls and test article

The test article, octamethylcyclotetrasiloxane (D4), was obtained from Sigma-Aldrich (St. Louis, MO). The negative control for the pituitary studies was culture media, and the positive control was sodium dodecyl sulfate (SDS), both provided by Sigma-Aldrich. 

### Fluorescent labeling solution for cell membranes

1,6-diphenyl 1,3,5- hexatriene (DPH) was purchased from Sigma Aldrich (St. Louis, MO). The DPH was prepared as a 10 mM stock solution in 100 % tetrahydrofuran (Sigma Aldrich, St. Louis, MO).

### Pituitary and hypothalamic cell culture

Primary rat pituitary cells were purchased from ScienCell (Carlsbad, CA) and cultured in the provided media. Cells were kept at 37 °C in a humidified incubator with 5 % CO_2_. The cells were maintained using standard cell culture techniques.

The Mouse Hypothalamic GnRH Neuronal Cell Line (GT1-7) was purchased from EMD Millipore (Burlington, MA). GT1-7 is an immortalized mature mouse hypothalamic GnRH neuronal cell line. Immortalized GnRH neurons were generated by introducing a transgene containing the promotor region of the GnRH gene coupled to the coding region of the SV40 T-antigen oncogene into transgenic mice. The resulting anterior hypothalamic tumors were removed from one of the mice, and the cells were dissociated and cloned. GT1-7 is a clonal line of mature differentiated GnRH neurons that exhibit high levels of GnRH mRNA and secrete GnRH in response to depolarization*. *This cell line is a suitable *in vitro* model for understanding GnRH-secreting neurons in the hypothalamus. GT1-7 cells were plated at 95,000 cells/well in a 24-well plate in DMEM (Gibco, Life Technologies, Carlsbad, CA) with 10 % FBS and 100 pM beta-estradiol. After 24 hr, the medium was changed to a phenol-free medium with 10 % charcoal-stripped serum. Test article and control mixtures (200 µL) were pre-incubated for 10 min at 37 °C with 5 % CO_2_ and then added to the cells. The cells were exposed to the test article or controls for 40 min, and then the cell culture medium was collected and stored at 4 °C for assessment of GnRH release. 

### Effects of D4 and SDS on pituitary cell membrane fluidity

Pituitary cell membrane exposures were performed by creating 2x stock solutions of sodium dodecyl sulfate (SDS) (Sigma Aldrich, St. Louis, MO) in phosphate-buffered saline (PBS) without calcium or magnesium, pH 7.4 (Gibco, Life Technologies, Carlsbad, CA) and D4 in PBS, 0.2 % Tween-20, 0.2 % Kolliphor (Sigma-Aldrich, St. Louis, MO), or 0.2 % absolute ethanol (Sigma-Aldrich, St. Louis, MO) to aid in solubilization of the D4. The 2x stock solutions were mixed to generate the final exposure concentrations of 30 %, 10 %, 3 %, 1 %, and 0.3 % D4 with 0.1 % absolute ethanol. SDS was prepared in culture media at final concentrations of 10 %, 3 %, 1 %, 0.3 %, and 0.1 %. The dosing solutions were added to the cells in the plates, and the plates were incubated on a plate shaker for 2, 10, or 15 minutes, depending on the study's phase. 

The pituitary cells were labeled for fluorescent polarization. A 10 mM stock of 1,6-diphenyl-1,3,5-hexatriene (DPH) was prepared in tetrahydrofuran by adding 0.0232g of DPH to 10 mL of tetrahydrofuran. To prepare the cell loading solution of DPH, a 10 µL aliquot of the 10 mM stock DPH was added to a 10 mL cell culture medium with 10 % FBS. After equilibration, the culture medium was removed and replaced with a medium containing 10 µM DPH, and the cells were incubated for 1 hr at room temperature with gentle shaking. Following the DPH loading phase, the medium was replaced with a medium without FBS containing D4 (0, 1 %, 3 %, 10 %, 30 %). Finally, cells were incubated for 15 minutes at room temperature. Fluorescent polarization was measured following this incubation using a blue spectrum fluorescence polarization cube Synergy H1M plate reader (BioTek, Winooski, VT).

### Effects of D4 and SDS on the mouse hypothalamic GnRH neuronal cell line (GR1-7)

Hypothalamus cell membrane exposures were performed by creating 2x stock solutions of SDS in PBS and D4 in PBS, 0.2 % Tween-20, 0.2 % Kolliphor (Sigma-Aldrich, St. Louis, MO), or 0.2 % absolute ethanol (Sigma-Aldrich, St. Louis, MO) to aid in solubilization of the D4. The 2x stock solutions were mixed to generate the final exposure concentrations of 30 %, 10 %, 3 %, 1 %, and 0.3 % D4 with 0.1 % absolute ethanol. SDS was prepared in culture media at final concentrations of 10 %, 3 %, 1 %, 0.3 %, and 0.1 %. The dosing solutions were added to the cells in the plates, and the plates were incubated on a plate shaker for 2, 10, or 15 minutes, depending on the study's phase. 

GT1-7 cells were seeded into a 96-well plate at a density of 35,000 cells/well. The cells were allowed to equilibrate overnight at 37^o^C with 5 % CO_2. _A 10 mM stock of 1,6-diphenyl-1,3,5-hexatriene (DPH) was prepared in tetrahydrofuran by adding 0.0232g of DPH to 10 mL of tetrahydrofuran. Following the DPH loading phase, the medium was replaced with a medium without FBS containing D4 (0, 0.1 %, 0.3 %, 1 %, 3 %, 10 %, or 30 %). The preparation, loading, and measurement of fluorescence polarization were the same as described above.

### GnRH and dopamine dosing dilutions

Dosing solutions of GnRH (Bachem, Bubendorf, Switzerland) were prepared by dissolving GnRH into a culture medium at a 25 mg/mL stock concentration. Stocks were diluted in culture media to the final exposure concentrations (0.000001, 0.00001, 0.0001, 0.001, 0.01, 0.1, 1, 10, 100, 250, 500, 1000 mg/mL). 

Dopamine was prepared as a 2x stock and diluted in 2x ascorbic acid media (DMEM culture media with a 2x physiologically relevant ascorbic acid concentration of 200 mM). Once the media was removed from the wells of the 96-well plates, 50 mL of 2x dopamine and 50 mL of 200 M ascorbic acid media were added to create the final dosing media with 100 mM ascorbic acid and 3, 10, 30, 100, 300, 1000 mM dopamine. Exposures were for 1,2, 4, 6, or 24 hours.

### GnRH release from the hypothalamus

GnRH release was monitored using Phoenix's LHRH/GnRH EIA kit (Burlingame, CA). The procedure followed was according to the kit protocol. Veratridine, potassium chloride (KCl), and Triton X-100 were purchased from Sigma-Aldrich and prepared in the same buffer as D4 for the hypothalamus studies. Kisspeptin was purchased from R&D Systems (Minneapolis, MN). The vehicle control was Hank's Balanced Salt Solution (HBSS), pH 7.4 (Lonza BioWhittaker, Chicago, IL). Veratridine and kisspeptin could be considered positive controls.

### LH and prolactin ELISAs

LH was measured in the culture medium with an LH ELISA kit from Enzo (Farmingdale, NY) and was performed according to the manufacturer's instructions. 100 mL of undiluted culture media was added to the wells, and no dilution was performed. Prolactin was assessed using the Rat RayBio@Prolactin ELISA Kit from RayBiotech (Norcross, GA). 100 µL of undiluted culture media was added to the wells for the primary pituitary cells. The kit was performed according to the manufacturer's instructions.

### Intracellular ATP 

Intracellular ATP was monitored using CellTiter Glo® Luminescent Cell Viability Assay (Promega). The assay was run according to the manufacturer's instructions. Following the exposure period, the media was removed by aspiration and replaced with 50 µL media plus 50 µL of lysis/reaction buffer (the lysis buffer contains all reactants necessary for the assay). The plates were placed on an orbital shaker and shaken for 10 min at room temperature. The luminescence signal was read with a BioTek (Santa Clara, CA) Synergy H1M Plate Reader in luminescence mode.

### Fluorescent polarization measurements

The fluorescence polarization measurements were made with a Synergy H1M plate reader from BioTek (Santa Clara, CA). The polarization, P, was evaluated by the relation, 

P = (I_p_-GI_v_)/(I_p_+GI_v_)

where I_p_ and I_v_ are the polarized fluorescence emission intensities measured at parallel and perpendicular geometry, respectively, to the direction of vertically polarized excitation light. The grating factor, G, is specific to the instrument and is defined by the ratio of I_p_ and I_v_. The ¯fluorescence anisotropy, r, was obtained from the relation, 

r = 2P/3-P

The microviscosity of the cell membranes was calculated by the Perrin equation (Shinitzky and Barenholz, 1974[26]), 

(r_0_)/r = 1 + C(r) x T X τ / η

where r_0_ =limiting fluorescence anisotropy (0.362 for DPH), C(r) = a parameter relating to the molecular shape and location of the transition dipoles of rotating fluorophore, T = absolute temperature, t = excited state lifetime (for DPH 11.4 ns), η = microviscosity of the medium. The variation of C(r) or τ for a system labeled with the same probe and temperature is considerably smaller than the variation of η. Therefore, the quantity (r_0_/r - 1)^-1 ^is the medium's apparent microviscosity. C(r) x T x τ = 2.4 poise

So, apparent microviscosity (η) poise = 2.4 r/0.362-r where r = 2P/3

### Statistical analysis 

The mean, standard of the mean, and standard error of polarization were calculated. Statistics were performed using a T-test with a P-value <0.05 being significant.

The optical density (OD) reads were background subtracted and normalized to the media control for the prolactin results. Data is shown as % inhibition of prolactin release with standard deviation and standard error of the mean calculated.

All data was compiled and organized in Excel. The mean, standard deviation, and standard deviation of the mean were calculated using Excel. All data were graphed using GraphPad Prism 9.

## Results

### Effect of D4 on primary pituitary cell membrane fluidity

**Figure 2:**
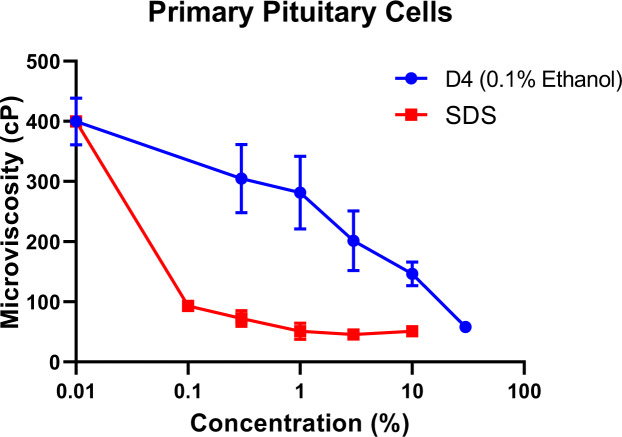
Apparent microviscosity (membrane fluidity) values of rat primary pituitary cell lines. 1,6-diphenyl 1,3,5- hexatriene (DPH) was dissolved in the primary pituitary cell membrane and then treated with various concentrations of SDS or D4, and fluorescence polarization was measured (fluorescence polarization is proportional to fluidity). Values represent mean + SEM of 3 wells per exposure concentration per treatment group.

Exposure of rat primary pituitary cells to increasing concentrations of SDS or D4 caused a dose-related decrease in the apparent microviscosity, i.e., an increase in the fluidity of cell membranes. SDS caused a 70.3 % decrease in microviscosity (Figure 2(Fig. 2)), while 30 % D4 caused a 66.7 % decrease. 

To ascertain whether decreased membrane microviscosity impacted cellular function, we assessed whether the GnRH pathway was functional in D4-treated cells by assessing LH and prolactin release from the cells in response to GnRH. GnRH was added to the cell culture medium at various concentrations (0.1 pg/mL to 1.0 mg/mL), and exposure times ranged from four to 24 hours. All conditions were examined in triplicate in 100 µL/wells in a 96-well plate. This resulted in no observable LH production by either negative control cells or those treated with D4 or the positive control (SDS) at concentrations that reduced membrane microviscosity (data not shown), possibly because the cell culture medium lacked specific essential components, such as calcium. 

**Figure 3:**
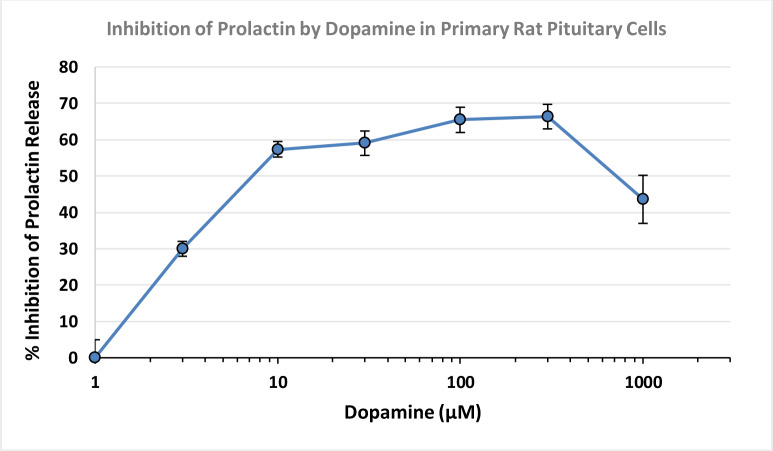
Prolactin Inhibition in primary pituitary cells exposed to increasing concentrations of dopamine. Twenty-four hours of incubation of the primary cells in the presence of dopamine resulted in a dose-dependent decrease in prolactin production. There was up to a 66.4 % decrease in prolactin production when dopamine (300 uM) was added. Values represent mean + SEM of 3 wells per exposure concentration.

The effect of D4 on prolactin release from the primary rat pituitary cells was assessed. Dopamine was added to the primary pituitary cell cultures at concentrations ranging from 3 mM to 1000 mM for 2, 6, or 24 hours. At 24 hours, prolactin was present at measurable concentrations in the cells grown with the medium only. Twenty-four hours of incubation of the cell culture with increasing concentrations of dopamine increased inhibition of the release of prolactin from the primary rat pituitary cells (Figure 3(Fig. 3)). There was a 65.5 % inhibition of prolactin release at 100 mM of dopamine, reaching an apparent plateau above this dopamine concentration. 

**Figure 4:**
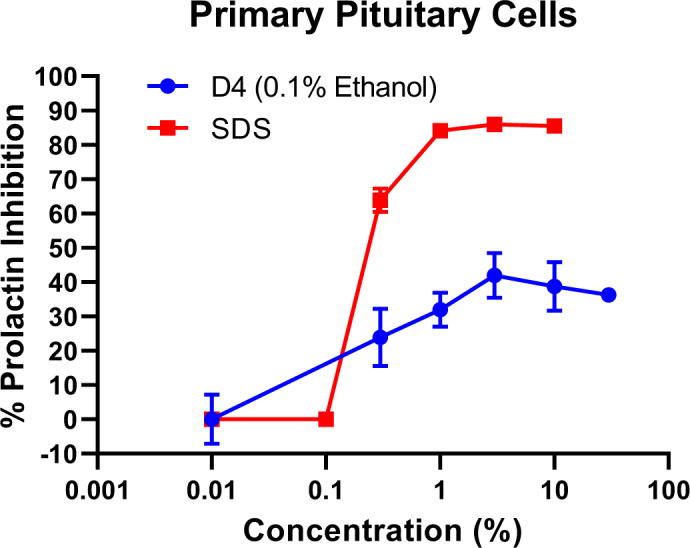
Prolactin Inhibition in primary pituitary cells exposed to increasing concentrations of D4. Both D4 and SDS inhibited prolactin secretion for the pituitary. D4's effect on prolactin inhibition is likely due to the alteration of membrane fluidity since D4 does not interact with dopamine receptors, per the work of Baker et al. (2010). Values represent mean + SEM of 3 wells per exposure concentration per treatment group.

Primary pituitary cells were incubated for 24 hours to achieve optimal prolactin release in the presence of 0 %, 0.3 %, 1.0 %, 3.0 %, 10.0 %, or 30.0 % D4 or with 0.0 %, 0.1 %, 0.3 %, 1.0 %, 3.0 % or 10.0 % SDS (Figure 4(Fig. 4)). Changes in prolactin release were assessed by ELISA. SDS substantially inhibited prolactin release, as evidenced by reduced prolactin levels in the culture media, from 63.9 % at 0.3 % SDS to 85.5 % at 10.0 % SDS. This is likely due, at least in part, to the detergent properties of SDS, which disrupt the lipid bilayer structure of cell membranes. Increasing concentrations of D4 inhibited prolactin release by up to 42.0 % in a dose-related manner, a somewhat similar decrease to that observed with SDS. The mode of action by which D4 inhibits prolactin release appears to be a non-specific effect of D4 on the fluidity of the cell membrane and not on dopamine receptor agonism since D4 is not a dopamine receptor agonist (Baker, 2010[4]). 

### Effect of D4 on GT1-7 hypothalamic cell membrane fluidity

Reduction of membrane viscosity may have indirect or non-specific effects on many cellular functions. For example, reduced microviscosity may affect the hypothalamus-pituitary axis's ability to regulate LH release, thereby indirectly impairing its vital role in the reproductive process in animals and humans. GnRH is critical in stimulating LH release from the pituitary. Several experiments were therefore conducted to investigate whether GnRH release and signaling pathways are impeded in mouse hypothalamic GT1-7 cell membranes exposed to concentrations of D4 that alter membrane microviscosity.

**Figure 5:**
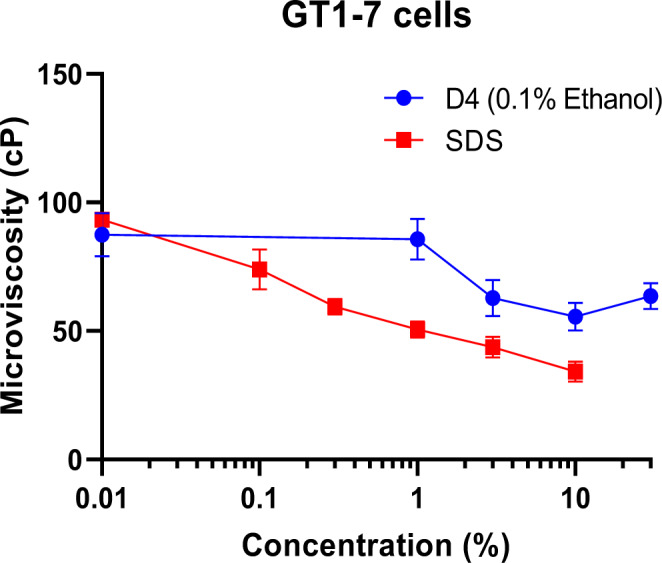
Effects of D4 on Membrane Fluidity in a Mouse Hypothalamic GT1-7 Cell Line. DPH was dissolved in the GT1-7 cell membrane and then treated with various concentrations of SDS or D4. Fluorescence polarization was measured (fluorescence polarization is proportional to fluidity). D4 produced a marked change in membrane fluidity at a concentration ≥2 %, as determined by a shift in polarization. Values represent mean + SEM of 3 wells per exposure concentration per treatment group.

Figure 5(Fig. 5) shows the effect of D4 and SDS on the microviscosity of the hypothalamic GT1-7 cells. GT1-7 cells seeded into 96-well plates at a density of 35,000 cells/well were preloaded with DPH and then exposed to D4 or SDS over a range of target concentrations. SDS reduced the microviscosity in a concentration-dependent manner from 95 cPoise (cP) to 30 cP. Similarly, D4 produced a concentration-related decrease in microviscosity. The microviscosity decreased from approximately 75 cP to 50 and 55 cP at a 10 % and 30 % D4 concentration, respectively. 

**Figure 6:**
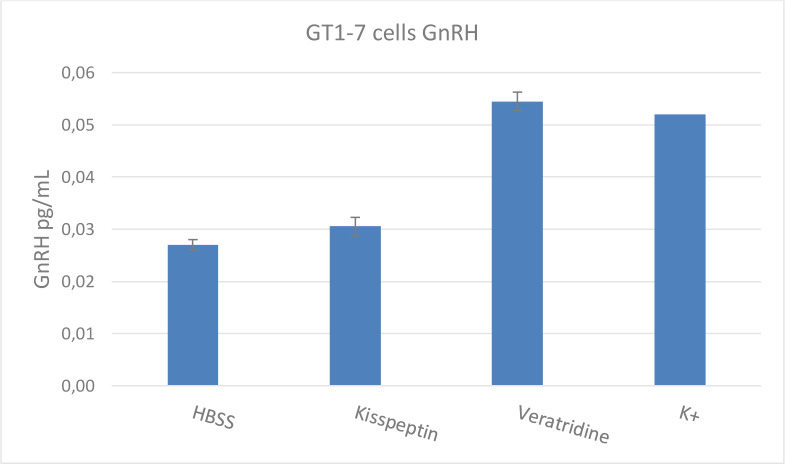
Effects of Veratridine, Kisspeptin, and Potassium Chloride on GnRH Release. GT1-7 cells were cultured and then treated with three agents (Veratridine (Ver (50 µM)), Kisspeptin (Kiss (100 nM)), and potassium chloride (59 mM)) known to stimulate GnRH release from the hypothalamus. This experiment demonstrates that the cell line responds to known stimulation to release GnRH. All treatment times were 40 min at 37^o^ C with 5 % CO2. Values represent mean + SEM of 3 wells per test material exposure.

In Figure 6(Fig. 6), GT1-7 cells were cultured, as described in the Materials and Methods section, and then treated with membrane depolarizing agents Veratridine (Ver) (50 µM) and potassium chloride (59 mM) and with Kisspeptin (100 nM), which binds to the GPR54 receptor (also known as Kiss1R) in the hypothalamus membrane. Depolarization of the test cells with high concentrations of KCl and subsequent release of GnRH was conducted as described by Sasson et al. (2006[25]). The use of Ver to induce membrane depolarization in rodent hypothalamus was conducted based on the work reported by Bourguignon et al. (1987[8]). This experiment showed that the cell line responds well to known stimulation to release GnRH (Figure 6(Fig. 6)).

**Figure 7:**
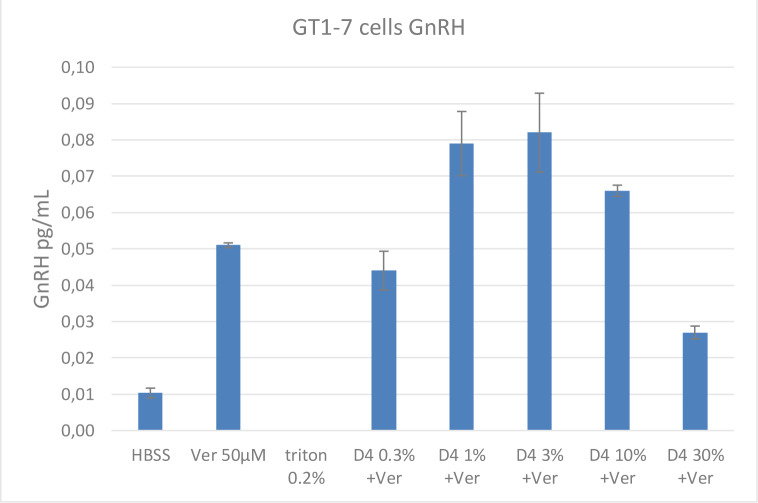
Effects of Veratridine and D4 on GnRH Release from GT1-7 Hypothalamus Cell Line. GnRH release from the hypothalamus cell line GT1-7 can be stimulated by veratridine (Ver). The figure above shows that 50 µM Ver caused a 5-fold increase in GnRH release. In the presence of D4 at 30 % theoretical exposure concentration, GnRH release was inhibited by nearly half. Values represent the mean + SEM of 3 wells per test material exposure.

To assess whether alterations in membrane fluidity by D4 can block the release of GnRH from the hypothalamus in the presence of agents that stimulate the release of GnRH, a second experiment was conducted using Ver and Ver with increasing concentrations of D4. Triton X-100, a membrane detergent, was used as a positive control. In Figure 7(Fig. 7), 50 µM of Ver caused a 5-fold increase in GnRH release. In the presence of D4 at 10 % and 30 % exposure concentrations, GnRH release was inhibited by nearly half. Triton X-100 inhibited the release of GnRH, as expected. 

**Figure 8:**
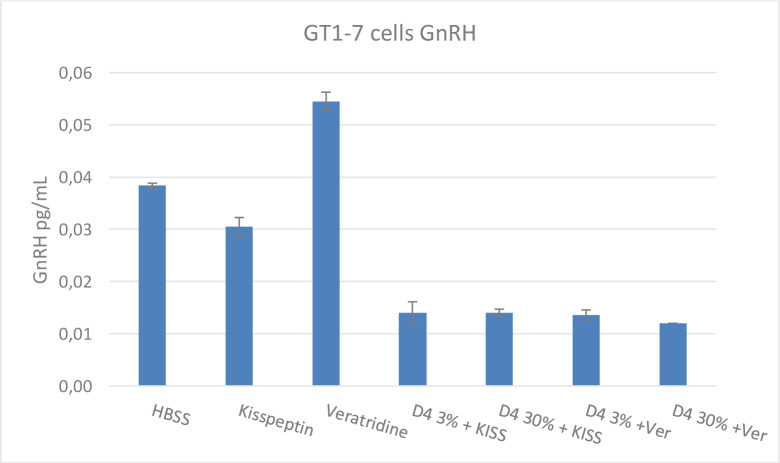
Effects Veratridine, Kisspeptin, and D4 on GnRH Release GT1-7 Hypothalamus Cell Line. Kisspeptin has been shown to stimulate the release of GnRH peptide from the hypothalamus by directly binding the Kiss receptor. A second experiment compared the effects of D4 on Kiss (100 nM) and Ver (50 µM) induced release of GnRH from the GT1-7 cells. In the presence of D4 at 3 % and 30 % exposure concentration, GnRH release was inhibited by nearly half. Values represent the mean + SEM of 3 wells per test material exposure.

Kisspeptin has been shown to stimulate the release of GnRH peptide from the hypothalamus by directly binding to the kisspeptin receptor (Messager et al., 2005[20]). An experiment was conducted that compared the effects of D4 on kisspeptin (100 nM) and Ver (50 µM) induced release of GnRH from the GT1-7 cells. Cells were cultured as described in the Materials and Methods section. In the presence of 3 %, 10 %, and 30 % D4, GnRH release from the GT1-7 cells was reduced by 67 % and 80 % in kisspeptin and veratridine-treated cells, respectively (Figure 8(Fig. 8)). 

**Figure 9:**
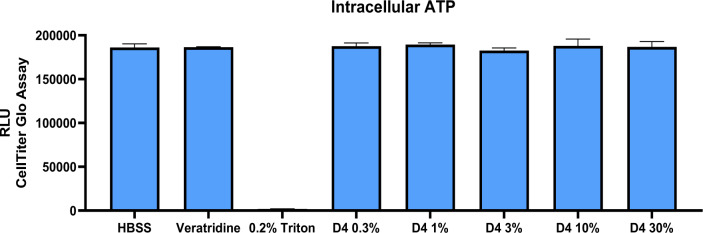
Effects of Test Compounds on Cell Viability. Cytotoxicity was measured using intracellular ATP as described in the materials and methods section. All treatment groups were compared to the vehicle control (HBSS). Treatments caused no measurable cytotoxicity under the conditions tested. The positive control (triton X) caused 100 % cell death. Values represent the mean + SEM of 3 wells per test material exposure.

To eliminate the possibility that the effects seen in these studies were due to cytotoxicity rather than changes in membrane fluidity, cytotoxicity studies were performed. Cytotoxicity was assessed by measuring intracellular ATP as described in the Materials and Methods section. All treatment groups were compared to the vehicle control Hank's Balanced Salt Solution. Treatment with ver or D4 ranging from 0.3 % to 30 % caused no measurable cytotoxicity under the conditions tested. Treatment with Triton X-100 caused 100 % cell death (Figure 9(Fig. 9)). 

## Discussion

In a two-generation reproductive study with rats, octamethylcyclotetrasiloxane caused a reduction in the number of corpora lutea, reductions in litter size resulting from a reduction in the number of implantation sites in the F_0 _and F_1_ generations, prolonged estrus cycles, and decreased mating and fertility indices in the F_1_ generation (Siddiqui et al., 2007[27]). The effects were attributed to exposure in females but not males (Meeks et al., 2007[18]). Meeks et al. (2007[18]) also demonstrated that the short period around ovulation was the critical exposure period for these reproductive effects. Follow-up studies with rats suggested inhibiting the pre-ovulatory surge of luteinizing hormone (LH), a predominant factor associated with reproductive effects (Quinn et al., 2007[22]). 

In rodents, the brain determines the timing of the preovulatory LH surge from the pituitary. It is triggered by a discharge of GnRH from the hypothalamus induced by a circadian neural signal that is coupled to the light-dark cycle and gated by an action (positive feedback) of estradiol in the preoptic area (POA) that, in part, is exerted on the population of kisspeptin neurons in the anteroventral periventricular nucleus (AVPV) (Plant, 2012[21]). Kisspeptin, a neuropeptide, is also crucial in controlling reproductive function by regulating gonadotropin-releasing hormone (GnRH) secretion (Plant, 2012[21]). Kisspeptin is synthesized in neurons in the hypothalamus, and interaction with the receptor GPR54 (also known as Kiss1R) is essential in regulating fertility. The expression and secretion of kisspeptin are influenced by circulating levels of sex steroids, including estradiol. When estradiol levels rise in the middle of the cycle, it stimulates kisspeptin production. This, in turn, acts on the kisspeptin receptor in GnRH neurons to promote the release of GnRH from the hypothalamus. The GnRH then acts on the anterior pituitary gland to release LH and follicle-stimulating hormone (FSH). The LH surge leads to ovulation, while FSH is important in the early stages of follicle development. Thus, kisspeptin is integral to the positive feedback loop in which rising estradiol levels lead to the mid-cycle LH surge. It acts as a critical upstream regulator of GnRH and thus indirectly influences LH release (Andersen, 2022[2]).

Prolactin is a hormone produced and secreted by the anterior pituitary gland, and it plays multiple roles in the reproductive system and other physiological processes. In female rats, prolactin is involved in several reproductive cycle stages, with its most prominent roles in ovulation, the luteal phase, and pregnancy. Prolactin plays a critical role in the regulation of ovulation in the rat. It has been shown that a surge in prolactin release from the anterior pituitary precedes the LH surge and the subsequent ovulation. 

It has been suggested that the mode of action for D4's reproductive effect in female rats is mediated through dopamine-like activity and, subsequently, an impact on prolactin secretion. However, D4 does not bind to the dopamine receptor, eliminating a direct agonist effect of D4 on prolactin secretion through the dopamine receptor (Baker, 2010[4]). Downstream effects on the dopamine signaling pathway cannot be excluded, but such effects may occur by mechanisms unrelated to direct interaction with components of the dopamine signaling pathway or with any other specific molecular effector site.

It also has been postulated that D4's reproductive effect in female rats is related to weak estrogenic activity. The potency of D4 compared to ethinylestradiol indicates that D4 is 585,000 times less potent than estrogen in the rat and 3.7 million times less potent than ethinylestradiol in the rat. It is implausible for D4 to have enough estrogenic activity to cause biological effects, including the reproductive effect seen in female rats (Borgert et al., 2018[7]; Matthews, 2021[17]; Andersen, 2022[2]). In humans, estrogen receptor occupancy could not be altered to a physiologically significant degree by estrogen receptor ligands possessing affinity for the estrogen receptor as low as that of D4, even at blood concentrations higher than those achieved in inhalation studies with D4 in rats (Borgert et al., 2024[6]). Since the reproductive effects of D4 in rats occur at exposure levels that overwhelm the ability of the test species to eliminate D4 (Borgert et al., 2025[5]), a specific mode of action through estrogen pathways is highly unlikely. 

 The results of the studies reported here support the hypothesis that D4 affects ovulation via a concentration-dependent, physical-chemical mode of action that is not specific for any particular component of the neuro-endocrine system and is, therefore, not endocrine disruption, but a non-specific effect. Sodium Dodecyl Sulfate (SDS) was used as the positive control in these studies. SDS is known to solubilize membranes by forming micelles that can incorporate lipid molecules from the membrane. Once the SDS exceeds critical micelle concentration, the surfactant solubilizes lipid molecules, leading to membrane disintegration (Rosen and Kunjappu, 1978[23]). D4 may behave differently due to the physical-chemical characteristics of D4. 

Alteration of the pituitary membrane fluidity inhibits prolactin release from the pituitary membrane, similar to dopamine. However, as noted above, D4 does not bind to the dopamine receptor, so this inhibition of prolactin secretion by D4 is most likely due to the alteration in the membrane fluidity of the pituitary membrane. Inhibition of prolactin secretion and a surge of prolactin secretion is necessary before the LH surge and subsequent ovulation. 

The hypothalamus is divided into several regions and nuclei, each with a specific role: a supraoptic region, which includes the supraoptic and paraventricular nuclei and is involved in the regulation of water balance and the secretion of antidiuretic hormone; tuberal region, which consists of the dorsomedial, ventromedial, and arcuate nuclei with the arcuate nucleus crucial for releasing hormones that regulate the anterior pituitary, and the mammillary region which contains the mammillary bodies which are involved in memory processing and autonomic function (Antoni, 1986[3]). The cell membranes of the hypothalamic cells are vital for various functions, including hormone secretion, which releases hormones directly into the bloodstream or the pituitary through exocytosis, a process dependent on the cell membrane and neural communication whereby hypothalamic neurons send and receive signals fundamental to their role in regulation homeostasis, including body temperature, hunger, thirst, and circadian rhythms (Alberts, 2002[1]). 

D4 and other chemicals with lipophilic properties appear to be capable of affecting the fluidity of biological membranes generally. The organs or tissues affected by alterations of membrane viscosity by a particular chemical likely depend on its specific kinetic properties, the route of exposure, the administered concentration or dose, and possibly other factors. The results shown here demonstrate that D4 increases the fluidity of membranes generally, including those of pituitary and fluidity of the hypothalamic (arcuate nuclei) origin. 

In cells of pituitary and hypothalamic origin, this alteration in membrane fluidity decreases the release of GnRH and kisspeptin. GnRH and kisspeptin are necessary for the pre-ovulatory LH surge from the pituitary (Sukhbaatar et al., 2013[28]). In the absence of a GnRH and kisspeptin release, there is no signal to the pituitary for the driver of the LH surge.

The structure and function of cells are critically dependent on membranes, which not only separate the interior of the cell from its environment but also define the internal compartments (Cooper, 2000[9]). Alterations in membrane fluidity effect, via an impact on the ease with which proteins may change conformation, the activity of various enzymes and transport systems. Early experiments have shown that toxic injury is frequently associated with modifications in plasma membranes' physical state and/or lipid composition (Le Grimellec et al., 1992[16]). Such changes in plasma membranes will likely play a role in cell dysfunction (Le Grimellec et al., 1992[16]). Several authors have shown that alterations in membrane fluidity can impact cellular functions, leading to non-specific effects or indirectly to toxicity (Meeks et al., 1981[19]; Jetten, et al., 1981[15], 1982[14]; Crews et al., 1983[10]). We have shown that D4 increases the membrane fluidity of cultured rat pituitary and hypothalamic cell membranes, which in turn inhibits the release of prolactin. The increase in hypothalamic cell membrane fluidity inhibits GnRH and kisspeptin release from the hypothalamus. The inhibition of prolactin, GnRH, and kisspeptin secretion would lead to an inhibition of the LH surge and ovulation since all three hormones are critical to normal ovulation. Furthermore, inhibition of the release of these three hormones through alteration of the pituitary and hypothalamic membrane fluidity would be considered physical-chemical and non-specific effects.

Functional in vitro studies of trans-membrane proteins after treatment with D4 were also pursued with HUVEC cells (Unpublished report, Iontox 2019[13]). Treating these cells with vascular endothelial growth factor (VEGF) leads to ERK1/2 phosphorylation. Cells were treated with increasing concentrations of VEGF and either D4 alone or combined with sunitinib maleate, a VEGF receptor inhibitor. D4 increased ERK1/2 phosphorylation compared to vehicle control at the highest concentration, and this increase was not affected by sunitinib, a potent VEGF inhibitor. It appears that D4 increases fluidity, i.e., a non-specific change in membrane packing, and these non-specific changes lead to enhanced ERK1/2 phosphorylation. Although it is difficult to accurately determine the aqueous phase exposure to D4 in these experiments due to the physical-chemical properties of D4, qualitatively, these results indicate that D4 at aqueous concentrations achievable in these mixed suspension experiments alters membrane integrity and membrane-associated biological functions. The results with HUVEC cells do not suggest that D4 caused specific alteration of ERK function but simply that D4 can change membrane fluidity and associated behaviors of membrane proteins/lipoproteins of various kinds via non-specific mechanisms (Unpublished Report, Iontox 2018[12], 2019[13]; Andersen, 2022[2]). 

In vitro studies require both technical replicates and biological replicates. Here, the requirement for technical replicates was met by measuring three wells per treatment group in each experiment and using the mean ± SEM as the response for each treatment group. The requirement for biological replicates was met by using three different cell lines, each from a different tissue and species: a primary rat pituitary cell line, a mouse hypothalamic GnRH neuronal cell line, and a human umbilical vein endothelial cell line (HUVEC). The impact of D4 on the membrane fluidity of all three cell lines was established unequivocally, as discussed in the text for the HUVEC cells and shown in the figures for rat pituitary cells and mouse hypothalamic neuronal cells. Measuring changes in membrane fluidity in three separate cell lines from different tissues and species fulfills the criterion for biological replication as well as, if not better than, conducting the same measurements in three separate experiments from the same cell line.

## Declaration

### Conflict of interest 

The authors of this paper have no conflicts of interest that affect the scientific analysis or conclusions. The Silicones Environmental, Health, and Safety Council supplied financial support for this paper. The analysis and views expressed in this paper are those of the authors.

### Artificial Intelligence (AI) - Assisted Technology

No artificial intelligence was used to conduct this work or prepare this manuscript.
